# Study of NAD-interacting proteins highlights the extent of NAD regulatory roles in the cell and its potential as a therapeutic target

**DOI:** 10.1515/jib-2022-0049

**Published:** 2023-03-07

**Authors:** Sara Duarte-Pereira, Sérgio Matos, José Luís Oliveira, Raquel M. Silva

**Affiliations:** IEETA/DETI, University of Aveiro, Aveiro, Portugal; Department of Medical Sciences, iBiMED – Institute of Biomedicine, University of Aveiro, Aveiro, Portugal; LASI – Intelligent Systems Associate Laboratory, Guimarães, Portugal; Universidade Católica Portuguesa, Faculty of Dental Medicine, Center for Interdisciplinary Research in Health (CIIS), Viseu, Portugal

**Keywords:** cancer, interactome, NAD metabolism, neurodegenerative disorders, signalling

## Abstract

Nicotinamide adenine dinucleotide (NAD) levels are essential for the normal physiology of the cell and are strictly regulated to prevent pathological conditions. NAD functions as a coenzyme in redox reactions, as a substrate of regulatory proteins, and as a mediator of protein-protein interactions. The main objectives of this study were to identify the NAD-binding and NAD-interacting proteins, and to uncover novel proteins and functions that could be regulated by this metabolite. It was considered if cancer-associated proteins were potential therapeutic targets. Using multiple experimental databases, we defined datasets of proteins that directly interact with NAD – the *NAD-binding proteins* (*NADBPs*) dataset – and of proteins that interact with NADBPs – the *NAD-protein–protein interactions* (*NAD-PPIs*) dataset. Pathway enrichment analysis revealed that NADBPs participate in several metabolic pathways, while NAD-PPIs are mostly involved in signalling pathways. These include disease-related pathways, namely, three major neurodegenerative disorders: Alzheimer’s disease, Huntington’s disease, and Parkinson’s disease. Then, the complete human proteome was further analysed to select potential NADBPs. TRPC3 and isoforms of diacylglycerol (DAG) kinases, which are involved in calcium signalling, were identified as new NADBPs. Potential therapeutic targets that interact with NAD were identified, that have regulatory and signalling functions in cancer and neurodegenerative diseases.

## 1 Introduction

Nicotinamide adenine dinucleotide (NAD) is a crucial metabolite in the cell, generally known for its function as cofactor in oxidation-reduction reactions responsible for energy production in the form of ATP, where it alternates between the oxidized (NAD+) and the reduced (NADH) forms. By transferring electrons between reactions, NAD participates in a multitude of metabolic processes that are key to the normal physiology of the cell including glycolysis, the citric acid cycle, fatty acids beta-oxidation and mitochondrial electron transport. Additionally, NAD is a substrate for proteins involved in cell survival, DNA damage repair, calcium signalling, or transcription regulation. NAD-dependent enzymes include sirtuins (SIRTs) [1], poly- and mono-(ADP-ribose) polymerases (PARPs and MARTs) [2, 3], and cyclic ADP-ribose hydrolases, such as CD38 [4]. Maintenance of NAD cellular levels depends on a balance between its production and its depletion, for which the interconversion between NAD+/NADH and NADP/NADPH is not accounted.

Another role for NAD was acknowledged more recently, where NAD would function as a direct modulator of protein–protein interactions (PPIs), through its binding to the NUDIX domain [5]. The NUDIX domain is a 23 amino acid long general structure of a **Nu**cleoside **Di**phosphate linked to a variable moiety **X**, with catalytic activity on nucleotides. Through their activity, many NUDIX proteins contribute to cellular homeostasis by cleaning the cell from deleterious compounds. Others regulate the concentrations of several metabolites, such as NAD, NADP and ADP-ribose. Others remove 5′-cap from RNA and control the stability of mRNA, as well as gene expression. Nevertheless, several NUDIX enzymes remain uncharacterized [6–8].

NAD binding to the NUDIX homology domain (NHD) of the Deleted in Breast Cancer 1 (DBC1) protein prevented its interaction with PARP1 [5], and the DBC1-PARP1 interaction inhibits PARP1 normal function in the DNA damage repair process. Conversely, DBC1 regulates the activity of several proteins such as the transcription factor p53; the androgen and estrogen receptors (AR and ER), that are involved in hormone signalling; the BRCA1, which is also a DNA damage repair protein; and other NAD-dependent proteins that are epigenetic regulators, such as SIRT1 and HDAC3 [9].

The PARP catalytic domain is an example of a conserved protein domain that is common to all proteins within the PARP family, in which resides their main function of transferring the ADP-ribose moiety from its substrate (NAD) to carboxylate groups of aspartic and glutamic residues [10].

In this study, we aimed to characterize the NAD interactome, due to multitude of NAD cellular functions and relevance of NAD metabolism in normal and pathological conditions. Considering the NAD role in regulating PPIs, we focused on NAD-binding proteins and their interactions. Multiple experimental databases were surveyed to define an NAD-binding dataset, that was characterized through pathway enrichment analysis and protein structural domains analysis. The full human proteome was then screened, and a selection of potential NAD-binding proteins were further analysed. As previously reported in [11], we identified new proteins that potentially interact with NAD. Here, we described in detail the NADBPs dataset, we predicted NAD interacting residues of known NADBPs to serve as a reference and we further analysed the NUDIX containing proteins. We also uncovered NADBPs that are cancer-associated and potential drug targets. In addition, we performed molecular docking to predict the NAD-binding to potential NADBPs.

## 2 Workflow

### 2.1 Data collection

#### 2.1.1 NAD-binding proteins (NADBPs) dataset

The first dataset was composed by proteins that directly interact with NAD, obtained from several databases of experimentally validated data. Namely, the following databases were searched: Human Metabolome Database (https://hmdb.ca/), STITCH (https://stitch4.embl.de/), Protein Data Bank (https://www.rcsb.org/), ChEMBL (https://www.ebi.ac.uk/chembl/), PubChem (https://pubchem.ncbi.nlm.nih.gov/) and DrugBank (https://go.drugbank.com/). The NADBPs dataset was composed by the sum of the proteins identified in the interactions from the six chemical databases used, as described in [11].

#### 2.1.2 NAD-related proteins dataset

To study the proteins potentially related to NAD, an NAD-related dataset was defined. This was made using “NAD” as a keyword search, which considered all proteins that have “NAD” in protein name or in any field of description, such as protein family names, gene description, function or ontology classification. All human reviewed proteins obtained through UniProt (https://www.uniprot.org/) [12] and from IMEX Consortium database (http://www.imexconsortium.org/) [13] were considered.

#### 2.1.3 NAD-protein–protein interactions (NAD-PPIs) dataset

A dataset composed by the proteins that interact with the NADBPs was built using three sources: BIOGRID (https://thebiogrid.org/) [14], STRING (https://string-db.org/) v.10 [15] and IMEX Consortium, as previously described [11].

### 2.2 Gene ontology (GO) analysis of the protein datasets

GO analysis was performed on PANTHER (http://pantherdb.org/) [16], through an overrepresentation test (Fisher’s exact, False Discovery Rate correction), using the Pathways annotation dataset (version 13.0). The NAD-binding, the NAD-related and the NAD-PPIs datasets were analysed.

### 2.3 Identification of putative NAD-binding proteins

The most frequent protein domains and protein families within the NADBPs dataset were identified [11]. The total of 20,303 human reviewed proteins from the Uniprot database were considered as a reference dataset, and the 50,588 unreviewed proteins as a test dataset. Proteins that presented at least one of the most frequent NADBPs domains were retrieved from both reference and test datasets. The genes/proteins that were found exclusively within the test dataset of unreviewed proteins were identified and further analysed using the NADbinder (http://crdd.osdd.net/raghava/nadbinder/) [17] to predict the number of NAD interacting residues, and the STRING database (https://string-db.org/) v. 11 [15], to obtain the interactions of each of those proteins.

### 2.4 Molecular docking

To evaluate the potential binding of NAD to the top target, an automated *in silico* molecular docking analysis was performed using SwissDock web server (http://www.swissdock.ch), as described by Grosdidier and collaborators [18]. NAD ligand was used as provided by ZINC database (https://zinc.docking.org/), with the ID ZINC8214766, and the protein 3D structures of the top target were retrieved from AlphaFold database (https://alphafold.ebi.ac.uk/) [19].

### 2.5 Cancer associated proteins and potential drug targets

Proteins from the NADBPs dataset were compared with catalogues of protein-coding genes from the subproteomes of the Human Protein Atlas (https://www.proteinatlas.org/) [20]. Namely, the cancer proteome, that contains a list of 569 mutated proteins strongly implicated in cancer, as defined through the catalogue of somatic mutations in cancer (COSMIC), and the druggable proteome, that contains a list of 754 proteins targeted directly by an FDA approved drug, were considered. Currently, approximately four thousand protein-coding genes in the UniProt database have experimental evidence of involvement in several disease conditions, including cancer, neurologic, systemic and cardiovascular disease. From those, a list of 1326 proteins annotated in The Human Protein Atlas as potential drug targets, was also considered, as they belong to known drug target protein classes, such as enzymes, transporters, receptors and ion-channels, and are not yet targets for FDA approved or experimental drugs in the Drugbank database.

## 3 Results

### 3.1 NAD-binding and NAD-related proteins differ in their predominant cellular roles

**Figure 1:**
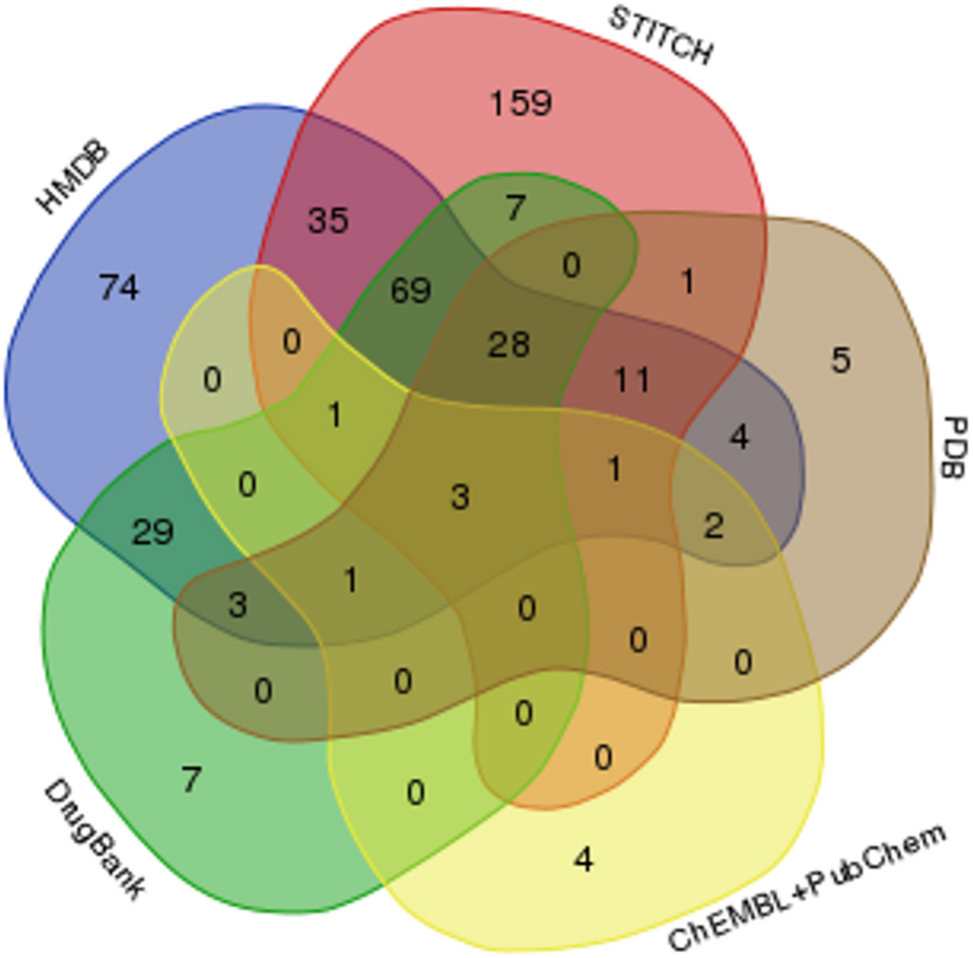
Venn-diagram showing the number of NAD-binding proteins obtained from each source.

After collecting data from six different databases, we obtained a NADBPs dataset composed by a total of 439 proteins (Figure 1 and Appendix A). The NAD metabolite was found under different forms and names, and both oxidized and reduced forms were included. The highest numbers of interactions with NAD were found on the databases STITCH, DrugBank and the Human Metabolome Database.

**Figure 2:**
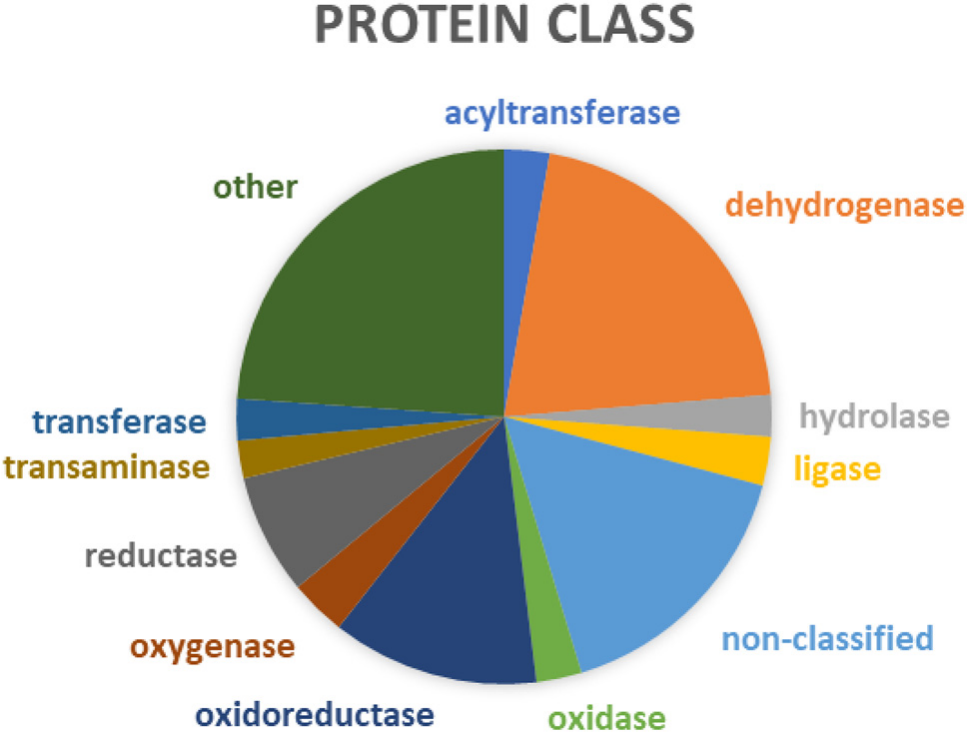
Classification of the NAD-binding proteins according to the protein class from PANTHER (www.pantherdb.org). The graphics represent the classes with more than 10 proteins. The remaining 43 classes comprising a total of 105 proteins are under the label “other” and 71 proteins remained non-classified.

The analysis of the 439 NADBPs showed that around 80% of these proteins were enzymes, most with catalytic activity, involved in metabolite interconversion. The major protein classes were dehydrogenases (92 proteins), from which over 30 were NADH dehydrogenase, and oxidoreductases (55 proteins), but several others were identified, as shown in Figure 2. More than one hundred proteins were mitochondrial isoforms of enzymes, which participate in the chain of reactions responsible for ATP production. Adding to enzymes that use NAD as cofactor in redox reactions, we also found all PARPs and all SIRTs, which are enzymes that use NAD as a substrate. Regarding their molecular function, a small number of proteins involved in regulation or transporter activities was also found.

The NAD-related datasets included all proteins potentially related to NAD, either by protein names or by any field of description. For the “NAD-related” dataset, we obtained 456 proteins from UniProt and 1907 from IMEX. In a total of 2125 proteins, only 238 were common to both sources. We then identified 279 proteins that were also present in the NADBP dataset, leaving a total of 1846 NAD-related proteins that do not bind NAD directly.

We performed a GO analysis on the 439 proteins of the NADBPs dataset (Table 1) and on the 1846 proteins of the NAD-related dataset, to compare the results of the enriched pathways obtained in each one. Only two pathways were common to the two datasets, Glycolysis and the FAS signalling pathway. We found 31 pathways specific of the NADBPs dataset, that were not enriched on the NAD-related dataset. Those included pathways related to biosynthesis or metabolism of nucleic acids, carbohydrates, and amino acids.

**Table 1:** NADBPs dataset pathway enrichment.

	Pathway	Fold enrichment
		(min. 3.04–max. 47.93)
**Biosynthesis**	Adrenaline and noradrenaline biosynthesis	6.18
	Alanine biosynthesis	47.93
	Androgen/estrogene/progesterone biosynthesis	23.97
	Asparagine and aspartate biosynthesis	35.95
	De novo purine biosynthesis	6.39
	Formyltetrahydroformate biosynthesis	29.96
	Gamma-aminobutyric acid synthesis	23.97
	Histidine biosynthesis	47.93
	Isoleucine biosynthesis	23.97
	Leucine biosynthesis	47.93
	Methionine biosynthesis	47.93
	O-antigen biosynthesis	19.17
	Proline biosynthesis	28.76
	Serine glycine biosynthesis	38.35
	Tetrahydrofolate biosynthesis	19.17
	Valine biosynthesis	31.95
**Basic metabolism**	5-Hydroxytryptamine degradation	38.35
	Acetate utilization	31.95
	Aminobutyrate degradation	47.93
	Fructose galactose metabolism	19.97
	Glutamine glutamate conversion	23.97
	Glycolysis	14.38
	Methylmalonyl pathway	19.17
	Phenylethylamine degradation	35.95
	Purine metabolism	20.54
	Pyruvate metabolism	30.5
	TCA cycle	38.35
	Xanthine and guanine salvage pathway	23.97
**Signaling**	Dopamine receptor mediated signaling pathway	4.79
	Endothelin signaling pathway	5.46
	FAS signaling pathway	7.05
	GABA-B receptor II signaling	11.28
	Heterotrimeric G-protein signaling pathway-Gi alpha and Gs alpha mediated pathway	3.04

In the NAD-related dataset, we found 36 pathways that did not appeared in the NADBPs dataset, that were mostly related to signalling. The highest fold enrichment values were found in the pentose phosphate pathway (the highest fold = 10.1), the JAK/STAT signalling, and four pathways related to p53 signalling. Of note, disease related pathways arose in the NAD related dataset, such as Alzheimer, Huntington, and Parkinson diseases. Also, signalling pathways related to angiogenesis, inflammation, and apoptosis, which are disease related mechanisms, were identified within the results.

### 3.2 Proteins that interact with NADBPs comprise about half of the human proteome

Then, the NAD-protein–protein interactions (NAD-PPIs), i.e., the proteins that interacted with the NADBPs were studied. Using the 439 proteins from the NADBPs dataset, 9823 pairs of proteins from STRING database were obtained, that corresponded to a total of 7815 unique gene name identifiers, 19,682 pairs from BIOGRID database, that corresponded to a total of 6479 unique gene IDs, and 5594 pairs from IMEX, that corresponded to a total of 3301 unique IDs. After mapping each type of ID retrieved from each database to the UniProtKB ID, with reviewed annotation (either using automatic tools or manually, in the case of automatically unmapped IDs), the duplicated entries we removed that were mainly due to gene or protein alternative names, or disease names associated to those genes. From STRING, a total of 7533 proteins were successfully mapped and 75 elements remained unmapped. From BIOGRID, a total of 5752 proteins were mapped and 54 remained unmapped. Most of these unmapped IDs were pseudogenes. From IMEX, 2500 proteins were mapped, and 90 elements remained unmapped. We found 40 CHEBI IDs, that were retrieved from CHEBI database for identification, but were not included for further analysis, since they corresponded to chemical compounds that interact with NADBPs and not protein-protein interactions, as it was intended. The proteins common to the three sources of PPIs were identified, and a final list of 10,020 proteins involved in PPIs with NADBPs remained.

**Figure 3:**
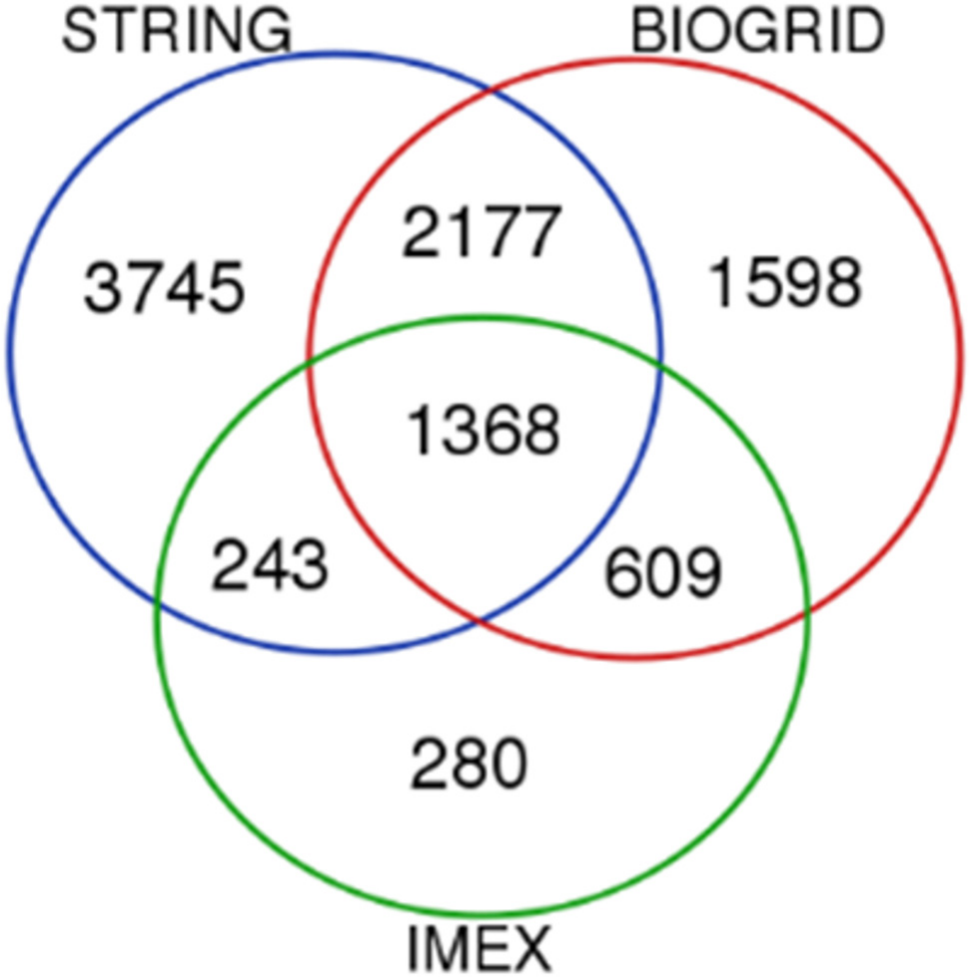
Venn-diagram representing the protein-protein interactions from STRING, BIOGRID and IMEX databases, where the 439 NADBPs were used as query.

As this represents about half of the human proteins annotated so far, according to the most recent version of UniProt Knowledge Database (UniProtKB 2020_06, [21]), with 20,379 reviewed proteins on the human proteome, the 1368 proteins common to all databases (Figure 3) were further analysed. With this, the selection of the most validated interactions was assured.

A GO analysis was performed on the 1368 proteins from the NAD-PPIs dataset and compared with the results from the NADBPs dataset described previously (Table 1). Similarly to the NAD-related dataset, the NAD-PPIs dataset presented an enrichment in several signalling pathways, as compared to the NADBPs dataset. The pathways with the highest number of genes (over 50) were related to hormone receptors signalling, namely for gonadotropin and for the gastrointestinal peptide hormones cholecystokinin and gastrin, followed by the Wnt signalling and angiogenesis pathways. Several other pathways were related to hormone or growth factor signalling, and disease pathways also emerged, namely three major neurodegenerative diseases, Alzheimer’s, Huntington’s, and Parkinson’s.

### 3.3 Overview of NADBPs protein structural domains

Protein domains analysis was performed on the 439 NADBPs through PFAM database and all matches that achieved an expectation value (E-value) below 1 (max. 0.88) were selected. The results show the top hit domain for each protein and how many hits were found. Within the 439 proteins, 1101 identifications were made, which corresponded to a total of 412 different domains. Two proteins didn’t have an identified domain (NDUFA11 and GPAT2) and, in the remaining 437 proteins, 222 different domains were identified as top hit. More than half of the proteins (56% – 247 proteins) belonged to the FAD/NAD(P)-binding Rossmann fold superfamily, and 27% belonged to the Ankyrin repeat superfamily. In our approach, the top 15 more common domains, which appeared in more than 10 proteins (Table 2), were selected. Five different ankyrin repeats were among these top domains found. Others were the short chain dehydrogenase, the aldehyde dehydrogenase family, the cytochrome P450 and the poly(ADP-ribose) polymerase (PARP) catalytic domain.

**Table 2:** The top 15 domains identified in the NADBPs dataset.

Domain hmm ID	Domain name	Number of proteins	Description
PF00023.29	Ank	30	Ankyrin repeat
PF13637.5	Ank_4	25	Ankyrin repeat
PF13857.5	Ank_5	24	Ankyrin repeat
PF00106.24	adh_short	23	Short chain dehydrogenase
PF13561.5	adh_short_C2	22	Enoyl-(Acyl carrier protein) reductase
PF13606.5	Ank_3	21	Ankyrin repeat
PF08659.9	KR	20	KR domain
PF12796.6	Ank_2	20	Ankyrin repeat
PF00211.19	Guanylate_cyc	18	Adenylate and Guanylate cyclase catalytic domain
PF00171.21	Aldedh	16	Aldehyde dehydrogenase family
PF00644.19	PARP	15	Poly(ADP-ribose) polymerase catalytic domain
PF01370.20	Epimerase	14	NAD dependent epimerase/dehydratase family
PF00153.26	Mito_carr	12	Mitochondrial carrier protein
PF00107.25	ADH_zinc_N	10	Zinc-binding dehydrogenase
PF00067.21	p450	10	Cytochrome P450

We also identified 65 proteins that had one of the 43 domains containing the term “NAD” in their names or descriptions. Eighteen proteins contain specifically one of the six different “NAD binding domain”, namely the D-isomer specific 2-hydroxyacid dehydrogenase, the 3-hydroxyacyl-CoA dehydrogenase, the lactate/malate dehydrogenase, the malic enzyme, the UDP-glucose/GDP-mannose dehydrogenase family, and the 6-phosphogluconate dehydrogenase NAD binding domains. The NUDIX domain was found only in two proteins from the NAD-binding dataset, namely NUDT12 and NUDT7.

### 3.4 Identification of 13 new NAD-binding proteins based on protein domains

We searched for the 15 domains that were identified in ten or more proteins from the NADBPs dataset (Table 2) within the dataset of the full human proteome unreviewed proteins (test dataset) and obtained 901 protein sequences. After removing all protein fragments and duplicates, 255 proteins were identified, which corresponded to 204 single genes. A similar approach was performed in the reference dataset yielding 474 genes. Given our aim to identify uncharacterized proteins, from the 204 genes, 195 that were also identified in the reference dataset were excluded and 8 genes remained, corresponding to 13 protein sequences, found uniquely in the test dataset (Table 3).

**Table 3:** Proteins identified from the test dataset.

UniProt ID	Status	Gene name	Protein name	Length	Number of NAD
					interacting
					residues
A0A087WV00	Unreviewed	DGKI	Diacylglycerol kinase (DAG kinase)	932	21
E7EM72	Unreviewed	DGKI	Diacylglycerol kinase (DAG kinase)	1047	29
E7EWQ4	Unreviewed	DGKI	Diacylglycerol kinase (DAG kinase)	1078	31
E9PFX6	Unreviewed	DGKI	Diacylglycerol kinase (DAG kinase)	734	23
E9PNL8	Unreviewed	DGKZ	Diacylglycerol kinase (DAG kinase)	707	26
E9PK39	Unreviewed	LRRK1	Leucine-rich repeat serine/threonine-protein kinase 1	650	12
E9PMK9	Unreviewed	LRRK1	Leucine-rich repeat serine/threonine-protein kinase 1	689	7
Q495V5	Unreviewed	POTEB	POTE ankyrin domain family member B (POTEB protein)	301	16
D6R9P2	Unreviewed	SLC9B2	Sodium/hydrogen exchanger 9B2	112	11
D6RC49	Unreviewed	TRPC3	Short transient receptor potential channel 3	276	5
J3QTB0	Unreviewed	TRPC3	Short transient receptor potential channel 3	793	33
A0A087WV96	Unreviewed	CYP3A7-CYP3A51P	CYP3A7-CYP3A51P readthrough	506	27
V9GXZ4	Unreviewed	FPGT-TNNI3K	FPGT-TNNI3K readthrough	949	24

Among the 13 proteins, there were five isoforms of the Diacylglycerol (DAG) kinase, four encoded by the DGKI gene, and one encoded by DGKZ gene. There were two other kinase isoforms, from the Leucine-rich repeat serine/threonine-protein kinase 1, encoded by the LRRK1 gene. There were also two proteins related to membrane transport, the Sodium/hydrogen exchanger 9B2 (SLC9B2) and two isoforms of a short transient receptor potential channel encoded by the TRPC3 gene. A smaller isoform of the POTEB member of the ankyrin family was also found. Of note, POTEB was the only protein that presented simultaneously two of the 15 domains (Ank_2 e Ank_5). Additionally, there were two proteins resultant from the readthrough of two genes, CYP3A7-CYP3A51P, which belong to a subfamily of the Cytochrome P450, and FPGT-TNNI3K, from the neighbouring fucose-1-phosphate guanylyltransferase (FPGT) and TNNI3 interacting kinase (TNNI3K) genes.

To evaluate the possibility that NAD has an impact on the interactions between these proteins, we further searched for the interactions of each of the proteins. DGKI and SLC9B2 had no reported interactions, as well as the proteins resultant from the two readthrough events. LRRK1 had the highest number of interactions, followed by TRPC3.

### 3.5 Number of NAD interacting residues in new and known NADBPs

The 13 identified proteins were further analysed using the NADbinder software (Table 3). Here, instead of the protein structure, the protein sequence is considered. The highest number of NAD-interacting residues was 33 and was identified in the longest isoform of TRPC3, with 793 amino acids, followed by the longest isoform of DGKI with 1078 amino acids, where 31 residues were identified. The five DAG kinase isoforms retrieved more than 20 NAD-interacting residues, as well as the two readthrough proteins. A positive correlation was observed between the amino acid length and the number of NAD-interacting residues identified.

To serve as a reference, six proteins known to be involved in NAD metabolism were additionally scanned for the number of NAD interacting residues. Namely, two enzymes that consume NAD intracellularly (PARP1 and SIRT1), two enzymes that consume NAD extracellularly (CD38 and CD73), and two enzymes that participate in NAD biosynthesis (NAMPT and NAPRT) were analysed. Among these sequences, there was no significant correlation between the number of NAD-binding residues and protein length. According to the NADbinder analysis results, CD73 had the highest number of NAD interacting residues (51), and the remaining NAD-consuming enzymes had between 37 and 43 residues, which were higher than the ones identified in the 13 previously studied proteins. NAMPT and NAPRT don’t interact directly with the NAD molecule, and presented 39 and 18 NAD interacting residues, respectively. However, they bind nicotinamide and nicotinic acid, similar molecules that are NAD precursors, and are responsible for the first steps of their conversion into NAD.

### 3.6 NUDIX containing proteins in NADBPs dataset

**Figure 4:**
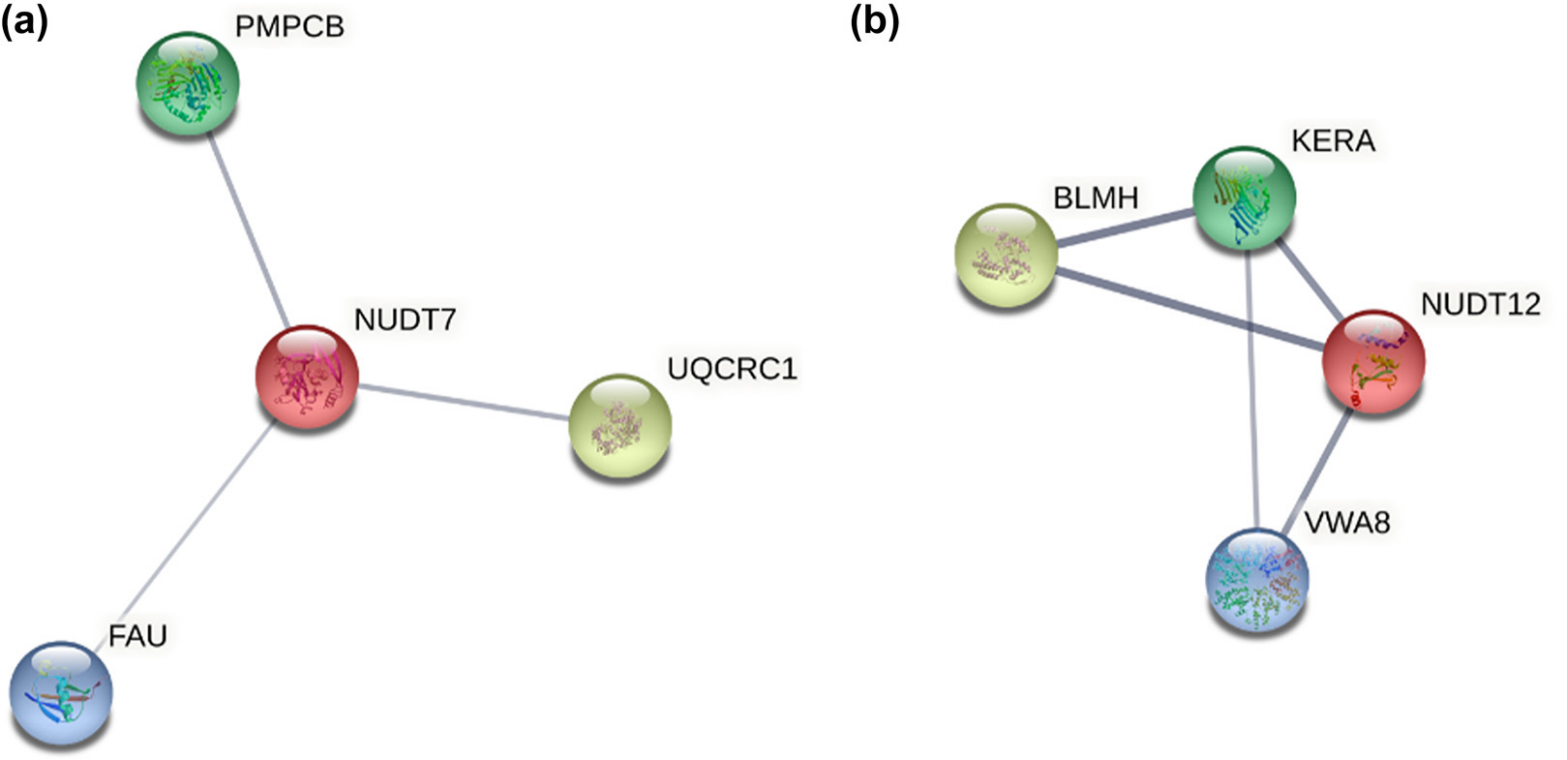
Protein–protein interactions of NUDIX proteins from the NAD-binding proteins dataset. (a) NUDT7 and (b) NUDT12. Queried proteins are represented by red nodes and the line thickness indicates the confidence level of the interaction. Only physical interactions are represented. The network was obtained through STRING (string-db.org).

As it was previously described that NUDIX domain directly interacts with NAD, the proteins within NADBPs dataset that are NUDIX hydrolases, NUDT7 and NUDT12, were also studied. In NUDT7, 21 NAD interacting residues were identified and, in NUDT12, only six residues were detected. We also searched for their interactors, considering only experimentally validated physical interactions, and found three proteins that interact with NUDT7 and three proteins that interact with NUDT12 (Figure 4).

### 3.7 Identification of NADBPS as potential new drug targets mutated in cancer

We found 122 proteins that are NAD-binding and potential drug targets. Two of them also belong to the set of cancer mutated genes, fumarate hydratase (FH) and 5′-nucleotidase, cytosolic II (NT5C2). Additionally, three other NADBPs, which mutations are implicated in cancer, were found in the catalogue of FDA approved targets, namely, 5-aminoimidazole-4-carboxamide ribonucleotide formyltransferase/IMP cyclohydrolase (ATIC), androgen receptor (AR) and isocitrate dehydrogenase 2 (IDH2).

## 4 Discussion

NAD binds to a large number of different proteins in order to perform a diversity of functions within the cell. In those reactions, NAD can: (1) act as an enzymatic cofactor in redox reactions, (2) be degraded by NAD-dependent enzymes, and (3) mediate protein-protein interactions, therefore regulating several cellular processes. Our approach in this study to identify potential NAD-binding proteins, drove us to a global analysis of the NAD interactome. We integrated data from various sources to include a large dataset of proteins that were already known to interact with the NAD molecule, or that were in some way related to NAD functions. We functionally characterized the protein datasets through gene ontology and protein structural domains analysis.

Through the analyses of enriched pathways, based on gene ontology annotations, we found that NADBPs are involved in a diversity of cellular pathways. The comparison with the NAD-related or the NAD-PPIs datasets emphasised that NADBPs are central in basic metabolism and biosynthetic processes. Nonetheless, essential metabolic pathways, such as glycolysis and TCA cycle, and signalling pathways mediated by GABA or dopamine receptors, were found in all datasets. Conversely, the proteins that participate in NAD-PPIs are involved in signalling pathways, from development and apoptosis to general immune and hormone responses, and including many disease pathways, showing the extension of the action of this small molecule.

Analysis of the protein structural domains showed that the ankyrin repeats were the most frequent, with some proteins presenting more than one ankyrin repeat in their structures. The ankyrin domain is very frequent in all human proteome as it mediates protein-protein interactions [22] and regulates the function of other proteins [23]. Confirming their high frequency, in the unreviewed dataset here obtained from the full human proteome, based on the UniProt database, 448 proteins have at least one of the five ankyrin repeats.

Adding to protein structural domains, the number of NAD interacting residues was considered, given that the direct binding of NAD at specific sites of a protein ultimately determines its action [17]. NAD binding to the NUDIX homology domain of DBC1 regulates its action on PARP1, by preventing the interaction between the two proteins [5]. In this study, no more than 10 residues were identified within the NUDIX domain that are conserved across several species. Considering the presence of a specific domain with a folding favourable to an interaction with a small molecule, only a small number of residues might be responsible for the actual interaction. The identification of NAD interacting residues within the sequence of known NADBPs, revealed that, while some NAD consuming enzymes had around 40 residues, the two NUDIX-containing domain proteins had lower numbers (21 and 6).

The role of the NAD-capped RNA hydrolase NUDT12 is directly associated with NAD, also known as deNADding enzyme, and it interacts with Bleomycin Hydrolase (BLMH) through the ankyrin repeats of NUDT12 [24]. The known role of the peptidase BLMH is to cleave the anti-cancer peptide Bleomycin, reducing the intracellular levels of the drug, but its primary biological function remains unknown.

**Figure 5:**
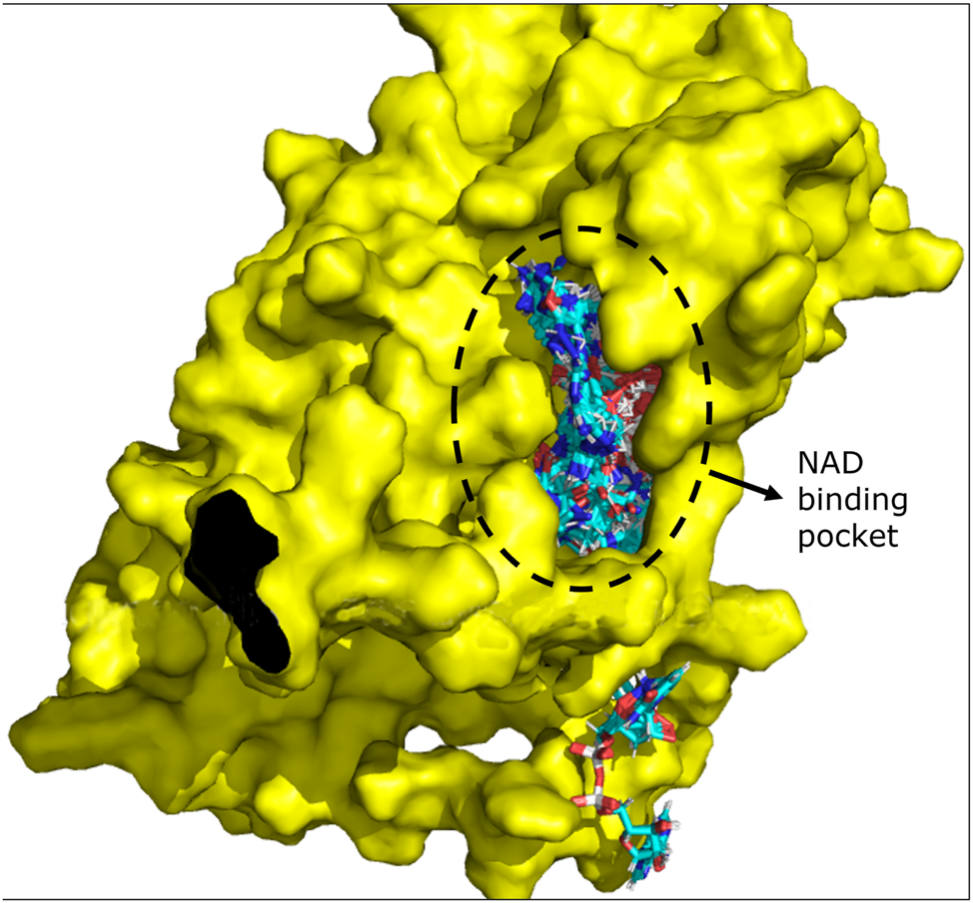
Docking results for NAD ligand on TRPC3 as a target (D6RC49 and J3QTB0). Protein 3D structures were obtained from AlphaFold (https://alphafold.ebi.ac.uk/) and visualized using Pymol software.

Among the new proteins that might potentially bind NAD identified in our study, TRPC3 (UniProt ID J3QTB0) had the ankyrin repeat domain and had the highest number of NAD-interacting residues. The molecular docking performed revealed a potential NAD-binding location on TRPC3. From a total of 31 clusters of docking positions obtained, 26 were placed within a same location, including the ones with the best scoring and lowest estimated energy (Figure 5).

The corresponding reviewed protein (UniProt ID: Q13507) of TRPC3 is longer than the two isoforms detected here, with 836 amino acids. Its known interactions were found to be mostly involved in signal transduction, response to stress, anatomical structure development, and transport processes, many of them related to calcium transport and signalling, such as the inositol trisphosphate (IP3) receptors ITPR1 and ITPR3, and the Sodium/calcium exchanger SLC8A1.

TRPC3 is a member of the transient receptor potential (TRP) channels family, which regulates intracellular calcium concentration [25] and is directly activated by lipids, specifically diacylglycerol (DAG). Together with IP3, DAG is a product of the hydrolysis of a phospholipid catalysed by the phospholipase C (PLC) enzymes. PLC gamma enzymes are key components of intracellular signalling, and some PLCG1 functions have been associated to a specific protein domain that directly interacts with TRPC3 and PLCG1, regulating calcium entry [26]. Very recently, the role of PLC gamma enzymes in disease development has been explored [27]. Of note, PLCG1 was also found in our dataset of NAD-PPIs, showing that it already binds other NADBPs, and several unreviewed isoforms of DAG kinases were identified in this study as potential NADBPs.

Both NAD-dependent signalling and calcium-dependent signalling are essential in the cell and therefore their dysregulation is often associated with disease. In particular, the role of NAD as a regulator of calcium channels has been recently reviewed, due to its impact on cancer treatment research [28], where calcium channels emerge as potential targets for anticancer therapy. In addition to cancer, the TRP channels, namely the TRPC3 group, regulate functions in neurons and are involved in various neurological and psychiatric disorders [29].

Interestingly, only one ion channel was identified in the primary NADBPs dataset in our study, named Transient receptor potential cation channel subfamily M member 2 (TRPM2). Although it was not identified in the domain analysis through Pfam, the presence of NUDIX domains in the structure of TRPM2 has been described in the literature and associated to its conformational changes and gating functions [30]. The activation of TRPM2 by NAD has been documented for over two decades and is one example of the relation between NAD and calcium metabolism [31].

In a final step of this research, we decided to investigate whether some of the NADBPs were potential therapeutic drug targets. We found FH and NT5C2, which are directly involved in NAD related reactions: the former participates in the TCA cycle and the latter in the NAD synthesis, specifically by catalysing the hydrolysis of NMN into NR or NAMN into NAR. Both enzymes are altered in cancer and are also associated with neurological diseases [32–34]. In addition, from the NADBPs dataset ATIC, AR and IDH2 are already being used as therapeutic targets. ATIC participates in purine biosynthesis, where it catalyses the last two steps of the pathway [35]. IDH2 is the mitochondrial isoform of the isocitrate dehydrogenases family of enzymes, that depends on NADP and calcium binding to perform the oxidative decarboxylation of isocitrate, one of the steps of the TCA cycle. Therefore, alterations in these enzymes will have an important impact in metabolism. IDH1 and IDH2 mutations have been described in different types of cancer, including glioblastoma, and are being targeted for acute myeloid leukaemia [36, 37]. The androgen receptor act as a transcription factor and, when activated by the hormone androgen, binds to target genes, and directly regulates gene transcription of a high number of genes. SIRT1, an NAD-dependent deacetylase, regulates AR activity, linking NAD metabolism to ligand-induced hormone signalling [38]. Aberrant expression of AR contributes to the progression of prostate cancer, making this protein a recognized therapeutic target in this context [39]. Alterations in AR have also been associated to neurological diseases, from developmental deficiencies to neurodegenerative disorders [40].

## 5 Conclusions

Concluding, this global study of the NAD interactome resulted in the identification of new potentially NAD-binding proteins, including TRPC3 and a few isoforms of DGA kinases, which are involved in calcium signalling. NADBPs participate in several metabolic pathways and signalling processes in the cell, while proteins interacting with NADPBs (NAD-PPIs) are mostly involved in signalling pathways. Furthermore, we identified NADBPs that are known (ATIC, AR and IDH2),as well as potential new drug targets in cancer (FH and NT5C2).

## Appendix A: List of the proteins that compose the NAD-binding proteins (NADBPs) dataset

**Table:**

UniProt ID	Gene name	Length	UniProt ID	Gene name	Length
Q8NSZO	AADAT	425	P30838	ALDH3A1	453
Q4L235	AASDH	1098	P51648	ALDH3A2	485
Q9NRN7	AASDHPPT	309	P43353	ALDH3B1	468
Q9UDR5	AASS	926	P48448	ALDH3B2	385
P80404	ABAT	500	P30038	ALDH4A1	563
P33897	ABCD1	745	P51649	ALDHSA1	535
P09110	ACAA1	424	Q02252	ALDH6A1	535
P42765	ACAA2	397	P49419	ALDH7A1	539
Q9UKU7	ACAD8	415	Q9H2A2	ALDH8A1	487
P28330	ACADL	430	P49189	ALDH9A1	494
P11310	ACADM	421	P04075	ALDOA	364
P16219	ACADS	412	P05062	ALDOB	364
P45954	ACADSB	432	P09972	ALDOC	364
P24752	ACAT1	427	Q96NU7	AMDHD1	426
Q9BWD1	ACAT2	397	P48728	AMT	403
Q8TDX5	ACMSD	336	P19801	AOC1	751
P21399	ACO1	889	O75106	AOC2	756
Q99798	ACO2	780	Q16853	AOC3	763
O14734	ACOT8	319	Q06278	AOX1	1338
P33121	ACSL1	698	P10275	AR	920
Q9NUB1	ACSS1	689	P62330	ARF6	175
Q9NR19	ACSS2	701	P15848	ARSB	533
Q08828	ADCY1	1119	P51689	ARSD	593
Q08462	ADCY2	1091	P54793	ARSF	590
O60266	ADCY3	1144	QSFYA8	ARSH	562
Q8NFM4	ADCY4	1077	QSFYB1	ARSI	569
O95622	ADCY5	1261	QSFYBO	ARS3	599
O43306	ADCY6	1168	Q6UVVY0	ARSK	536
P51828	ADCY7	1080	P51690	ARSL	589
P40145	ADCY8	1251	P52961	ART1	327
O60503	ADCY9	1353	Q13508	ARTS	389
P07327	ADH1A	375	Q93070	ART4	314
P00325	ADH1B	375	Q96L15	ARTS	291
P00326	ADH1C	375	A6ND91	ASPDH	283
P08319	ADH4	380	P31939	ATIC	592
P11766	ADHS	374	O75936	BBOX1	387
P28332	ADH6	368	P54687	BCAT1	386
P40394	ADH7	386	O15382	BCAT2	392
Q63HM1	AFMID	303	P12694	BCKDHA	445
P23526	AHCY	432	P21953	BCKDHB	392
O43865	AHCYL1	530	Q02338	BDH1	343
Q96HN2	AHCYL2	611	Q9BUT1	BDH2	245
O95831	AIFM1	613	Q93088	BHMT	406
P14550	AKR1A1	325	P53004	BLVRA	296
P15121	AKR1B1	316	P30043	BLVRB	206
O60218	AKR1B10	316	Q10588	BST1	318
Q04828	AKR1C1	323	Q13137	CALC00O2	446
P52895	AKR1C2	323	P04040	CAT	527
P42330	AKR1C3	323	Q8N4T8	CBR4	237
P17516	AKR1C4	323	P28907	CD38	300
P51857	AKR1D1	326	Q16878	CD01	200
P00352	ALDH1A1	501	P28329	CHAT	748
O94788	ALDH1A2	518	Q8NE62	CHDH	594
P47895	ALDH1A3	512	P21964	COMT	271
P30837	ALDH1B1	517	P43155	CRAT	626
Q3SY69	ALDH1L2	923	Q9UKG9	CROT	612
P05091	ALDH2	517	Q9Y2S2	CRYL1	319
O75390	CS	466	PO4406	GAPDH	335
Q9Y600	CSAD	493	O14556	GAPDHS	408
Q13363	CTBP1	440	O75600	GCAT	419
P56545	CTBP2	445	Q92947	GCDH	438
P32929	CTH	405	P23434	GCSH	173
P00167	CYBSA	134	Q9Y2T3	GDA	454
Q9UHQ9	CYBSR1	305	Q13630	GFUS	321
Q6BCY4	CYBSR2	276	P23378	GLDC	1020
P00387	CYB5R3	301	P00367	GLUD1	558
Q7L1T6	CYBSR4	521	P49448	GLUD2	558
Q6IPT4	CYBSRL	315	O60547	GMDS	372
Q53TN4	CYBRD1	286	P49915	GMPS	693
P08574	CYC1	325	P17174	GOT1	413
P99999	CYCS	105	Q8NHS2	GOT1L1	421
P05093	CYP17A1	508	P00505	GOT2	430
Q02318	CYP27A1	531	Q9HCL2	GPAM	828
P05181	CYP2E1	493	Q6NUI2	GPAT2	795
Q6VVX0	CYP2R1	501	Q86UL3	GPAT4	456
Q9NYLS	CYP39A1	469	P21695	GPD1	349
Q02928	CYP4A11	519	Q8N335	GPD1L	351
QSTCH4	CYP4A22	519	P43304	GPD2	727
P22680	CYP7A1	504	Q9UBQ7	GRHPR	328
O75881	CYP7B1	506	P00390	GSR	522
Q9UNU6	CYP8B1	501		H3C1	
P11182	DBT	482		H3C2	
Q16698	DECR1	335		H3C3	
O15121	DEGS1	323		H3C4	
Q9UBM7	DHCR7	475		H3C6	
P00374	DHFR	187	P68431	H3C7	136
Q86XFO	DHFR2	187		H3C8	
P49366	DHPS	369		H3C10	
Q96HY7	DHTKD1	919		H3C11	
P10515	DLAT	647		H3C12	
P09622	DLD	509	O95479	H6PD	791
P36957	DLST	453	Q9U383	HACL1	578
Q9NRD9	DUOX1	1551	Q16836	HADH	314
Q9NRD8	DUOX2	1548	P40939	HADHA	763
P30084	ECHS1	290	P55084	HADHB	474
P42126	ECI1	302	P42357	HAL	657
Q08426	EHHADH	723	P31937	HIBADH	336
Q9GZV4	EIFSA2	153	Q6NVY1	HIBCH	386
Q9NXB9	ELOVL2	296	P35914	HMGCL	325
Q9NYP7	ELOVLS	299	PO4035	HMGCR	888
Q8TC92	ENOX1	643	P09601	HMOX1	288
Q16206	ENOX2	610	P30519	HMOX2	316
P22413	ENPP1	925	P15428	HPGD	266
O14638	ENPP3	875	P28845	HSD11B1	292
O95864	FADS2	444	P80365	HSD11B2	405
Q8WVX9	FAR1	515	P14061	HSD17B1	328
Q96K12	FAR2	515	Q99714	HSD17B10	261
Q96IV6	FAXDC2	333	Q8NBQS	HSD17B11	300
P37268	FDFT1	417	Q53GQ0	HSD17B12	312
P07954	FH	510	Q9BPX1	HSD17B14	270
P98177	FOX04	505	P37059	HSD17B2	387
Q6ZNAS	FRRS1	592	P37058	HSD17B3	310
Q14376	GALE	348	P51659	HSD17B4	736
P07902	GALT	379	O14756	HSD17B6	317
P56937	HSD17B7	341	O15239	NDUFA1	70
Q92506	HSD17B8	261	O95299	NDUFA10	355
P14060	HSD3B1	373	Q86Y39	NDUFA11	141
P26439	HSD3B2	372	Q9UI09	NDUFAl2	145
Q9H2F3	HSD3B7	369	Q9P0J0	NDUFA13	144
P48735	IDH2	452	O43678	NDUFA2	99
P50213	IDH3A	366	O95167	NDUFA3	84
O43837	IDH3B	385	O00483	NDUFA4	81
P51553	IDH3G	393	Q9NRX3	NDUFA4L2	87
P20839	IMPDH1	514	Q16718	NDUFAS	116
P12268	IMPDH2	514	P56556	NDUFA6	128
Q9NPH2	ISYNA1	558	O95182	NDUFA7	113
O15229	KMO	486	P51970	NDUFA8	172
Q16719	KYNU	465	Q16795	NDUFA9	377
Q9H9P8	L2HGDH	463	O14561	NDUFAB1	156
P00338	LDHA	332	O75438	NDUFB1	58
Q6ZMR3	LDHAL6A	332	O96000	NDUFB10	172
Q9BYZ2	LDHAL6B	381	Q9NX14	NDUFB11	153
P07195	LDHB	334	O95178	NDUFB2	105
P07864	LDHC	332	O43676	NDUFB3	98
Q86WU2	LDHD	507	O95168	NDUFB4	129
O00214	LGALS8	317	O43674	NDUFB5	189
P01229	LHB	141	O95139	NDUFB6	128
P18858	LIG1	919	P17568	NDUFB7	137
P49916	LIG3	1009	O95169	NDUFB8	186
P49917	LIG4	911	Q9Y6M9	NDUFB9	179
P21397	MAOA	527	O43677	NDUFC1	76
P27338	MAOB	520	O95298	NDUFC2	119
P40925	MDH1	334	P28331	NDUFS1	727
P40926	MDH2	338	O75306	NDUFS2	463
P48163	ME1	572	O75489	NDUFS3	264
P23368	ME2	584	O43181	NDUFS4	175
Q16798	ME3	604	O43920	NDUFSS	106
Q15800	MSMO1	293	O75380	NDUFS6	124
P00156	MT- CYB	380	O75251	NDUFS7	213
P11586	MTHFD1	935	O00217	NDUFS8	210
P13995	MTHFD2	350	P49821	NDUFV1	464
Q9H903	MTHFD2L	347	P19404	NDUFV2	249
P42898	MTHFR	656	P56181	NDUFV3	108
P49914	MTHFS	203	Q9HAN9	NMNAT1	279
Q13613	MTMR1	665	Q9BZQ4	NMNAT2	307
Q13614	MTMR2	643	Q96T66	NMNAT3	252
Q9Y217	MTMR6	621	Q9NWW6	NMRK1	199
Q9Y216	MTMR7	660	Q9NPI5	NMRK2	230
P03886	MT-ND1	318	P40261	NNMT	264
P03891	MT-ND2	347	Q13423	NNT	1086
P03897	MT-ND3	115	P15559	NQO1	274
P03905	MT-ND4	459	P16083	NQO2	231
P03901	MT-ND4L	98	Q15738	NSDHL	373
P03915	MT-NDS	603	P49902	NTSC2	561
P03923	MT-ND6	174	Q9BQG2	NUDT12	462
Q99707	MTR	1265	P00O24	NUDT7	238
O95544	NADK	446	Q6D104	NXN	435
Q4GON4	NADK2	442	Q04671	OCA2	838
Q6IA69	NADSYN1	706	Q02218	OGDH	1023
P43490	NAMPT	491	Q9ULDO	OGDHL	1010
Q6XQN6	NAPRT	538	P55809	OXCT1	520
Q53GL7	PARP10	1025	Q9NRC8	SIRT7	400
Q9NR21	PARP11	338	P53007	SLC25A1	311
Q9HOJ9	PARP12	701	Q9UBX3	SLC25A10	287
Q460N5	PARP14	1801	Q02978	SLC25A11	314
Q460N3	PARP15	678	Q9BQT8	SLC25A21	299
Q8N5Y8	PARP16	322	O14975	SLC27A2	620
Q9UGNS	PARP2	583	Q9Y2P5	SLC27A5	690
Q9Y6F1	PARP3	533	Q9NTN3	SLC35D1	355
Q9U10C3	PARP4	1724	Q8N808	SLC35G3	338
Q2NL67	PARP6	630	Q9Y6L6	SLCO1B1	691
Q8N3A8	PARP8	854	Q9NPDS	SLCO1B3	702
Q8IXQ6	PARP9	854	P84022	SMAD3	425
P11498	PC	1178	Q13485	SMAD4	552
P61457	PCBD1	104	Q00796	SORD	357
Q9HONS	PCBD2	130	Q9UHE8	STEAP1	339
P05165	PCCA	728	Q8NFT2	STEAP2	490
P05166	PCCB	539	Q658P3	STEAP3	488
P35558	Pau	622	Q687X5	STEAP4	459
P08559	PDHA1	390	P08842	STS	583
P29803	PDHA2	388	Q9P2R7	SUCLA2	463
P11177	PDHB	359	P53597	SUCLG1	346
O00330	PDHX	501	Q96I99	SUCLG2	432
P00558	PGK1	417	Q8NBK3	SUMF1	374
P07205	PGK2	417	P17735	TAT	454
O43175	PHGDH	533	O15178	TBXT	435
Q9NRX4	PHPT1	125	O95455	TGDS	350
Q9POZ9	PIPDX	390	Q7Z3E1	TIPARP	657
P00491	PNP	289	O95271	TNKS	1327
P01189	POMC	267	Q9H2K2	TNKS2	1166
O43272	PRODH	600	P60174	TPI1	249
Q9Y617	PSAT1	370	P51580	TPMT	245
Q14914	PTGR1	329	O94759	TRPM2	1503
Q8N8N7	PTGR2	351	Q86TN4	TRPT1	253
Q8TE99	PXYLP1	480	P14679	TYR	529
P32322	PYCR1	319	P17643	TYRP1	537
Q96C36	PYCR2	320	O60701	UGDH	494
Q53H96	PYCR3	274	Q16851	UGP2	508
P09417	QDPR	244	Q9UDW1	UQCR10	63
Q15274	QPRT	297	P14927	UQCRB	111
P11233	RALA	206	P31930	UQCRC1	480
Q8IZVS	RDH10	341	P22695	UQCRC2	453
O75452	RDH16	317	P47985	UQCRFS1	274
Q92781	RDHS	318	P07919	UQCRFI	91
Q6NUM9	RETSAT	610	O14949	UQCRQ	82
P12271	RLBP1	317	Q96N76	UROC1	676
Q16518	RPE65	533	Q8NBZ7	UXS1	420
O75845	SCSD	299	P47989	XDH	1333
Q8NBX0	SCCPDH	429			
P22307	SCP2	547			
Q8N3Y7	SDR16C5	309			
P34896	SHMT1	483			
P34897	SHMT2	504			
Q96EB6	SIRT1	747			
Q81)06	SIRT2	389			
Q9NTG7	SIRT3	399			
Q9Y6E7	SIRT4	314			
Q9NXA8	SIRTS	310			

## References

[1] Haigis MC, Sinclair DA (2010). Mammalian sirtuins: biological insights and disease relevance. Annu Rev Pathol.

[2] Gupte R, Liu Z, Kraus WL (2017). PARPs and ADP-ribosylation: recent advances linking molecular functions to biological outcomes. Genes Dev.

[3] Bütepage M, Eckei L, Verheugd P, Lüscher B (2015). Intracellular mono-ADP-ribosylation in signaling and disease. Cells.

[4] Chini EN (2009). CD38 as a regulator of cellular NAD: a novel potential pharmacological target for metabolic conditions. Curr Pharmaceut Des.

[5] Li J, Bonkowski MS, Moniot S, Zhang D, Hubbard BP, Ling AJ (2017). A conserved NAD+ binding pocket that regulates protein-protein interactions during aging. Science.

[6] Carreras-Puigvert J, Zitnik M, Jemth AS, Carter M, Unterlass JE, Hallström B (2017). A comprehensive structural, biochemical and biological profiling of the human NUDIX hydrolase family. Nat Commun.

[7] Kulikova VA, Nikiforov AA (2020). Role of NUDIX hydrolases in NAD and ADP-ribose metabolism in mammals. Biochemistry (Mosc).

[8] McLennan AG (2006). The Nudix hydrolase superfamily. Cell Mol Life Sci.

[9] Santos L, Colman L, Contreras P, Chini CC, Carlomagno A, Leyva A (2019). A novel form of Deleted in breast cancer 1 (DBC1) lacking the N-terminal domain does not bind SIRT1 and is dynamically regulated in vivo. Sci Rep.

[10] Amé JC, Spenlehauer C, de Murcia G (2004). The PARP superfamily. Bioessays.

[11] Duarte-Pereira S, Matos S, Oliveira JL, Silva RM, Fdez-Riverola F, Rocha M, Mohamad MS, Caraiman S, Gil-González AB (2022). The NAD interactome, identification of putative new NAD-binding proteins. Practical Applications of Computational Biology and Bioinformatics, 16th International Conference (PACBB 2022).

[12] The UniProt Consortium (2017). UniProt: the universal protein knowledgebase. Nucleic Acids Res.

[13] Orchard S, Kerrien S, Abbani S, Aranda B, Bhate J, Bidwell S (2012). Protein interaction data curation: the International Molecular Exchange (IMEx) consortium. Nat Methods.

[14] Stark C, Breitkreutz BJ, Reguly T, Boucher L, Breitkreutz A, Tyers M (2006). BioGRID: a general repository for interaction datasets. Nucleic Acids Res.

[15] Szklarczyk D, Gable AL, Lyon D, Junge A, Wyder S, Huerta-Cepas J (2019). STRING v11: protein-protein association networks with increased coverage, supporting functional discovery in genome-wide experimental datasets. Nucleic Acids Res.

[16] Mi H, Muruganujan A, Huang X, Ebert D, Mills C, Guo X (2019). Protocol Update for large-scale genome and gene function analysis with the PANTHER classification system (v.14.0). Nat Protoc.

[17] Ansari HR, Raghava GP (2010). Identification of NAD interacting residues in proteins. BMC Bioinf.

[18] Grosdidier A, Zoete V, Michielin O (2011). SwissDock, a protein-small molecule docking web service based on EADock DSS. Nucleic Acids Res.

[19] Jumper J, Evans R, Pritzel A, Green T, Figurnov M, Ronneberger O (2021). Highly accurate protein structure prediction with AlphaFold. Nature.

[20] Uhlén M, Fagerberg L, Hallström BM, Lindskog C, Oksvold P, Mardinoglu A (2015). Proteomics. Tissue-based map of the human proteome. Science.

[21] Consortium U (2019). UniProt: a worldwide hub of protein knowledge. Nucleic Acids Res.

[22] Barz MJ, Hof J, Groeneveld-Krentz S, Loh JW, Szymansky A, Astrahantseff K (2020). Subclonal NT5C2 mutations are associated with poor outcomes after relapse of pediatric acute lymphoblastic leukemia. Blood.

[23] Li J, Mahajan A, Tsai MD (2006). Ankyrin repeat: a unique motif mediating protein-protein interactions. Biochemistry.

[24] Kumar A, Balbach J (2021). Folding and stability of ankyrin repeats control biological protein function. Biomolecules.

[25] Wu H, Li L, Chen KM, Homolka D, Gos P, Fleury-Olela F (2019). Decapping enzyme NUDT12 partners with BLMH for cytoplasmic surveillance of NAD-capped RNAs. Cell Rep.

[26] Samanta A, Hughes TET, Moiseenkova-Bell VY (2018). Transient receptor potential (TRP) channels. Subcell Biochem.

[27] Wen W, Yan J, Zhang M (2006). Structural characterization of the split pleckstrin homology domain in phospholipase C-gamma1 and its interaction with TRPC3. J Biol Chem.

[28] Liu Y, Bunney TD, Khosa S, Macé K, Beckenbauer K, Askwith T (2020). Structural insights and activating mutations in diverse pathologies define mechanisms of deregulation for phospholipase C gamma enzymes. EBioMedicine.

[29] Yu P, Cai X, Liang Y, Wang M, Yang W (2020). Roles of NAD+ and its metabolites regulated calcium channels in cancer. Molecules.

[30] Huang Q, Wang X, Lin X, Zhang J, You X, Shao A (2020). The role of transient receptor potential channels in blood-brain barrier dysfunction after ischemic stroke. Biomed Pharmacother.

[31] Wang L, Fu TM, Zhou Y, Xia S, Greka A, Wu H (2018). Structures and gating mechanism of human TRPM2. Science.

[32] Sano Y, Inamura K, Miyake A, Mochizuki S, Yokoi H, Matsushime H (2001). Immunocyte Ca2+ influx system mediated by LTRPC2. Science.

[33] Schmidt C, Sciacovelli M, Frezza C (2020). Fumarate hydratase in cancer: a multifaceted tumour suppressor. Semin Cell Dev Biol.

[34] Duarte RRR, Bachtel ND, Côtel MC, Lee SH, Selvackadunco S, Watson IA (2019). The psychiatric risk gene NT5C2 regulates adenosine monophosphate-activated protein kinase signaling and protein translation in human neural progenitor cells. Biol Psychiatr.

[35] Vergis JM, Beardsley GP (2004). Catalytic mechanism of the cyclohydrolase activity of human aminoimidazole carboxamide ribonucleotide formyltransferase/inosine monophosphate cyclohydrolase. Biochemistry.

[36] Biedermann J, Preussler M, Conde M, Peitzsch M, Richter S, Wiedemuth R (2019). Mutant IDH1 differently affects redox state and metabolism in glial cells of normal and tumor origin. Cancers.

[37] Cerchione C, Romano A, Daver N, DiNardo C, Jabbour EJ, Konopleva M (2021). IDH1/IDH2 inhibition in acute myeloid leukemia. Front Oncol.

[38] Houtkooper RH, Cantó C, Wanders RJ, Auwerx J (2010). The secret life of NAD+: an old metabolite controlling new metabolic signaling pathways. Endocr Rev.

[39] Tan MH, Li J, Xu HE, Melcher K, Yong EL (2015). Androgen receptor: structure, role in prostate cancer and drug discovery. Acta Pharmacol Sin.

[40] Bianchi VE, Rizzi L, Bresciani E, Omeljaniuk RJ, Torsello A (2020). Androgen therapy in neurodegenerative diseases. J Endocr Soc.

